# Right cervical lymphadenopathy: a rare presentation of metastatic hepatocellular carcinoma

**Published:** 2014

**Authors:** Irappa Madabhavi, Apurva Patel, Mukesh Choudhary, Asha Anand, Harsha Panchal, Sonia Parikh

**Affiliations:** Department of Medical and Pediatric Oncology, GCRI, Ahmedabad, Gujarat, India

**Keywords:** Metastatic, Hepatocellular carcinoma, Cervical lymphadenopathy

## Abstract

Hepatocellular carcinoma (HCC) is the most common, malignant tumor of the liver. Hepatocellular carcinoma (HCC) commonly metastasizes to the lungs, abdominal lymph nodes, adrenal glands, or bones. Distant lymph node metastases are rare in hepatocellular carcinoma. A 49-year-old male patient presented with right sided neck mass. On examination there was right cervical lymphadenopathy and hepatomegaly. Excisional cervical lymph node biopsy showed metastatic carcinoma. However, further examination of the biopsy specimen for immuno-histochemistry markers, shows positivity for HepPar-1 & CD-10 suggestive of hepatocellular carcinoma. Considering the high incidence of HCC in Asia, a special attention should be given to such unusual site of presentation and metastasis of HCC; therefore, not to miss the diagnosis.

## Introduction

Hepatocellular carcinoma (HCC) is the most common, malignant tumor of liver. HCC is associated with hepatitis C virus (HCV), hepatitis B virus (HBV), other hepatitis viruses, autoimmune hepatitis, steatohepatitis, primary biliary and sclerosing cholangitis, intake of aflatoxin-contaminated food, diabetes, and obesity (1). However, major risk factor for hepatocellular carcinoma in developing countries is mainly chronic hepatitis B and C. Cervical lympadenopathy is a common clinical problem. The differential diagnosis includes benign and malignant causes. Benign causes are bacterial, viral, tubercular and parasitic infections and sarcoidosis. Malignant causes are leukemia, lymphoma, metastasis from head and neck, lung, breast, abdominal and pelvic organ and testicular malignancies. Left supraclavicular lymphadenopathy (Virchow's node) may be the first sign of lung, breast, abdominal and testicular malignancies (2). Metastasis of HCC to the cervical and supraclavicular lymph node scarcely reported and very few case reports are available in the literature. Here, we report a case of HCC that presented as right cervical lymphadenopathy.

## Case Reports

**Figure 1:**
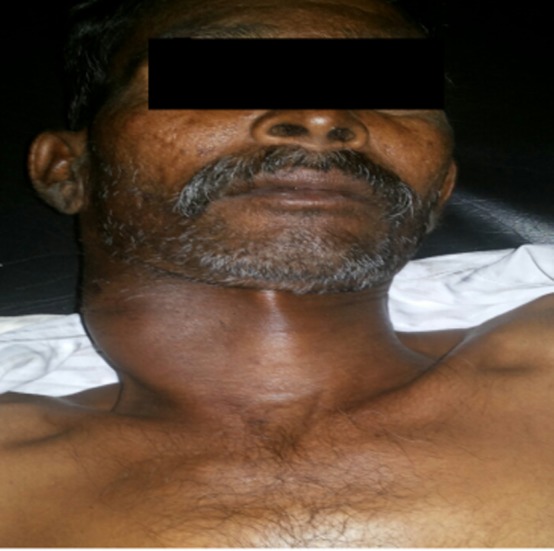
This figure is showing cervical nodal mass i.e. Soft, non-tender not fixed to the underlying tissue and mass was 4.0 x 3.0 cm in size.

A 49-year-old male patient, with ECOG performance status of 1, admitted for evaluation of progressive right-sided neck mass, loss of weight, appetite and general weakness of 3 months duration. There was no history of pain over the mass, fever, cough, chest pain, jaundice, night sweats and any lesion at head and neck region. He was non-alcoholic, non-smoker and non-diabetic. Patient had no history suggestive of any drug intake or jaundice in the past or any immune compromised status and his family history was not contributory.On admission, his temperature, pulse rate, respiratory rate and blood pressure were within the normal range. On examination right cervical nodal mass was noticed which was soft in consistency, non-tender, not fixed to the underlying tissue and not associated with any discharge from local site. Size of the mass was around 4.0 x 3.0 cm in size (Figure 1).

There was no icterus, pallor, pedal edema, palmar erythema, spider angioma, gynecomastia or testicular atrophy. However, supraclavicular axillary and inguinal lymph nodes were not palpable. On per abdominal examination, liver margin was palpated 5 cm below the right costal margin in midclavicular line and there was no ascites. 

Complete blood count and renal functions were within normal limit. Serum Alanine aminotransferase (ALT), Aspartate transaminase (AST), gamma glutamyl transpeptidase (GGT), Lactate Dehydrogenase (LDH), Ferritin and prothrombin time (PT) levels were within normal limits. Mild hypoalbuminemia (serum albumin; 2.14 g/dl) was present. Serum alpha-fetoprotein (1.69 ng/ml) was within normal limits. Patient serology was positive for Hepatitis B surface antigen and HBV-DNA. Antibody to hepatitis C virus (anti-HCV) and PCR for HCV-RNA for hepatitis C, antimitochondrial antibody and carcinoembryonic antigen (CEA) were negative.

**Figure 2:**
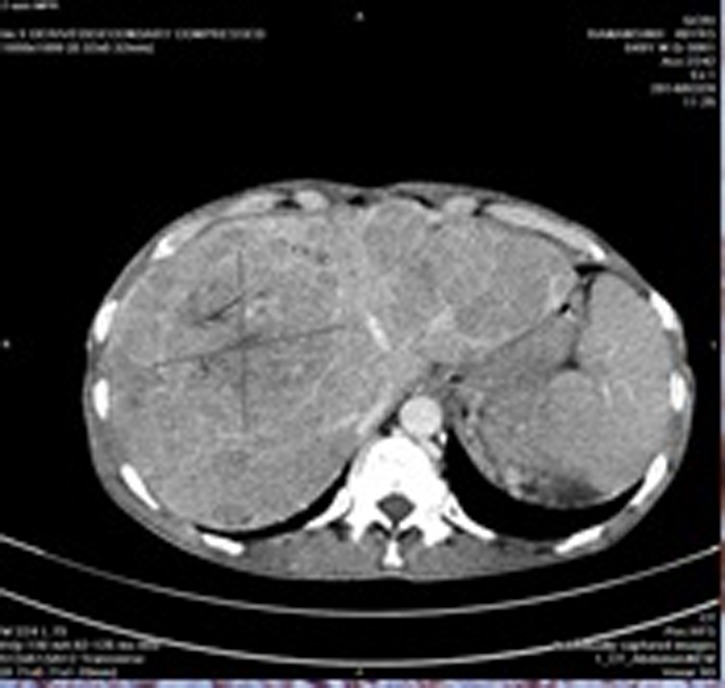
Abdomen CT showing heterogeneous echo texture lesions in right lobe of liver, measuring 83.8 x 84.6 mm in size.

Computerized tomography (CT) scan examination of neck was revealed presence of 3x4x4.2 cm size heterogeneous echo texture lesion in right level II and III. Abdominal CT scan shows hepatomegaly measuring about 17 cm in span and shows multiple heterogeneous echo texture lesions in both lobes of liver, largest measuring 83.8x84.6 mm in size (Figure 2) and multiple hypo echoic nodes noted in periportal, peripancreatic, celiac, paraaortic and iliac region.

**Figure 3:**
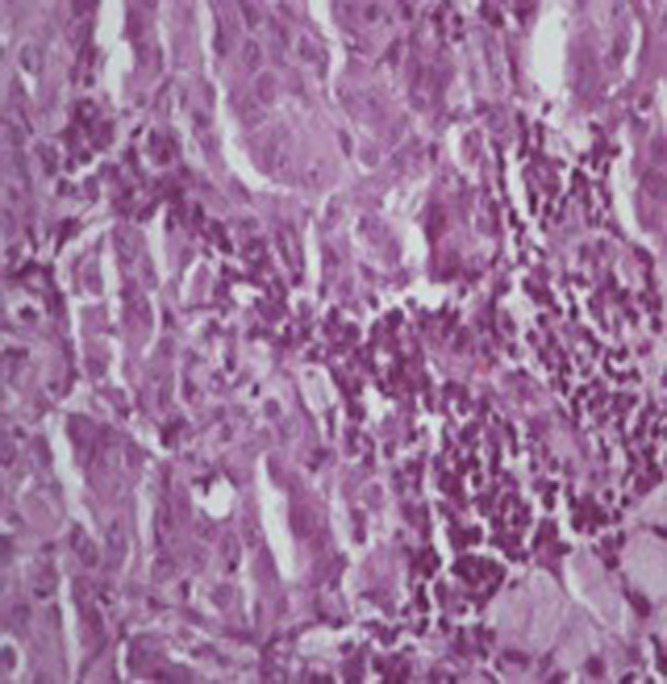
showing cells arranged in sinusoidal pattern and individual cells are polygonal in shape have clear to eosinophilic abundant cytoplasm & hyper chromatic nucleus and prominent nucleoli.

Presence of 116.2x84.1 mm size heterogeneously enhancing soft tissue density lesion was noted in left supra renal region that lesion infiltrates upper pole of left kidney and 82x58.8 mm size lesion also noted in right suprarenal region that was suggestive of metastasis. The patient underwent cervical lymph node biopsy which shows cells arranged in sinusoidal pattern and individual cells are polygonal in shape have clear to eosinophilic abundant cytoplasm & hyper chromatic nucleus and prominent nucleoli (Figure 3).

**Figure 4:**
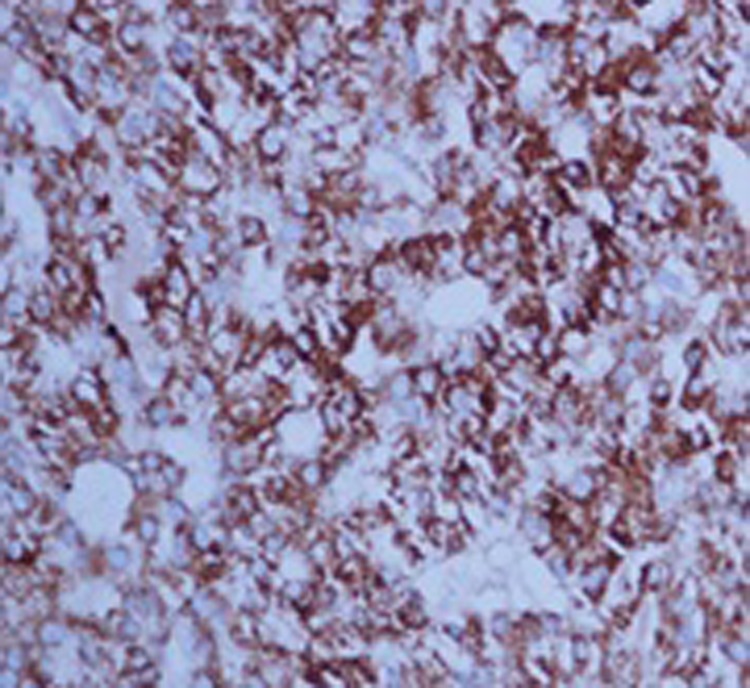
Showing Hep Par 1 positivity.

**Figure 5:**
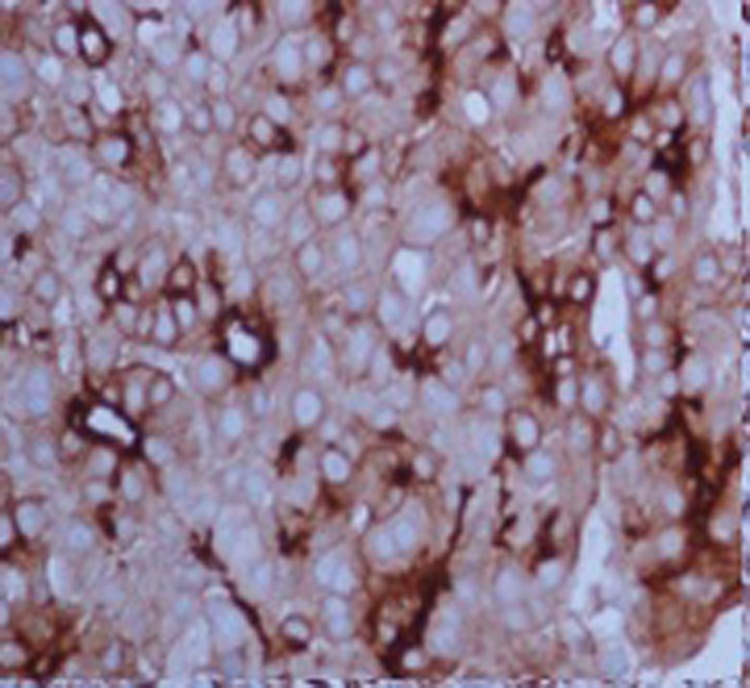
Showing CD 10 positivity.

On further examination tumor cells had strong immunoreactivity for hepatocyte paraffin 1 (HepPar 1) (Figure 4) and CD 10 (Figure 5) but negative for TTF-1, CEA, ALK-1, LCA, vimentin, S-100 and chromogranin. The results of immunohistochemistry were thus consistent with a diagnosis of metastatic hepatocellular carcinoma.

Patient was started on sorafenib 400mg twice a day and is under treatment till date. After one month of starting the sorafenib there was significant improvement in the performance status and symptoms in the form of increased appetite, increased weight and subjective wellbeing of the patient.

**Figure 6:**
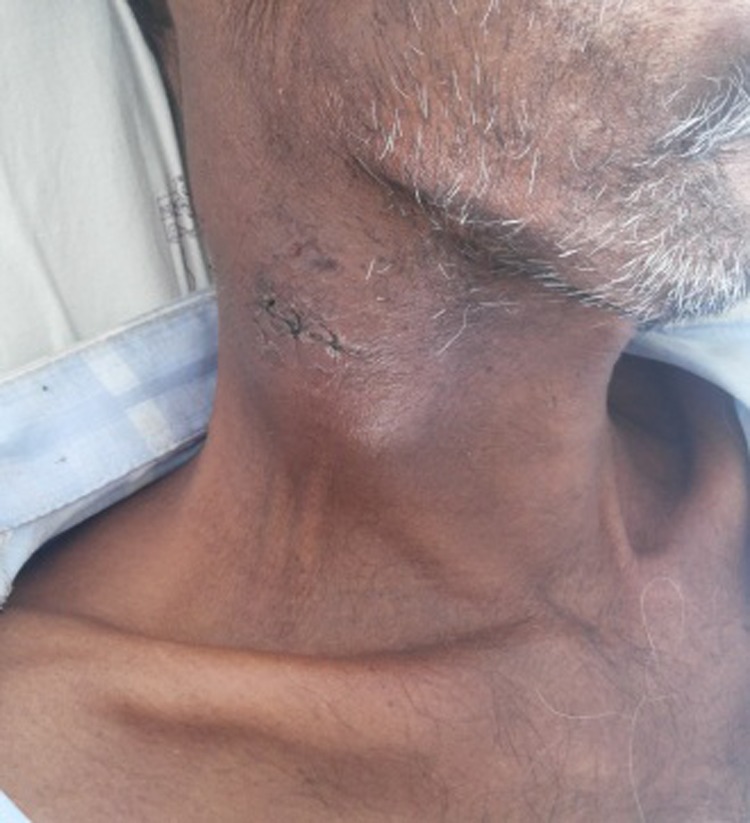
Showing regression of nodal mass after 1 month of sorafenib.

There was also decrease in the size of liver mass and the neck nodes (Figure 6). Imaging studies were suggestive of decrease in the size of neck and liver mass. Patient is under regular check up in our centre for clinical signs and signs of liver decompensation and imaging studies with 2 monthly dynamic CT abdomen for any progression of the disease since 7 months

## Discussion

Hepatocellular carcinoma (HCC) represents the sixth most common malignancy and the third most common cause of cancer-related death in both developed and developing countries. It is usually seen in the sixth and seventh decades of life in the western world, whereas in sub-saharan Africa and northeast Asia, it usually occurs earlier, in the fourth decade and more common due to a high prevalence of chronic HBV infection (3). It is found more commonly in the males. The course of clinically apparent disease is rapid and if untreated most patients die within three to six month of diagnosis.

HCC shows both intra hepatic and extra hepatic metastasis; however, intra hepatic metastasis appears to occur more frequently. Metastasis of HCC occurs by three main routes i.e. blood, direct spread, or lymphatic dissemination. About 30-50% patients of HCC show extra hepatic metastasis at the time of first presentation. Distant metastases occur from the HCC most predominantly to the lungs (55%), followed by abdominal lymph nodes (41%), adrenal glands and bones (28%) (4).

Hematogenous metastasis is more common than lymphatic metastasis. Lymphatic metastasis occurs according to normal anatomical pathways so lymphatic metastases often involve regional nodes; rarely distant nodes. At autopsy lymphatic metastasis is seen in 25% to 40% of HCC patients; however, clinically lymphatic metastasis is less common (5). 

There are three types of lymphatic vessels of liver depending on their locations: portal, sublobular and superficial or capsular. Sublobular and superficial lymphatic vessels are responsible for regional and distant (abdominal, mediastinal, axillary, supraclavicular, and cervical) lymph nodes metastasis. Sublobular lymphatic vessels lead into lymphatic vessels running along the inferior vena cava. Superficial lymphatic vessels have dense network and travel through the falciform ligament, the triangular ligament and coronary ligament either towards the diaphragm or porta hepatis or regional lymph nodes (6, 7).

Distant lymph node metastases are rare in hepatocellular carcinoma but mainly seen in left supraclavicular lymph nodes probably via the hepatic node and then through the thoracic duct. Few cases are reported that show right supraclavicular lymph node metastasis (8-10) and only few case reports have been reported with right cervical lymphadenopathy (11, 12).

One possible explanation of unusual distant lymph node metastasis is that lymphatic vessels and lymph production increases in patients with HCC. Because HCC expressing vascular endothelial growth factor-C that lead to lymphangiogenes and these creates complex bypasses of lymphatic flow that’s account for distant lymph nodes metastasis (6). Another explanation is that in few people; the right subclavian and jugular lymphatic trunks to from a right lymphatic duct; which drains into the junction of right internal jugular and subclavian veins (10).

The diagnosis of metastatic HCC in extra hepatic site can be made easily with immunohistochemical markers. Commonly use immunohistochemical (IHC) markers are Hep Par 1, polyclonal carcinoembryonic antigen, glypican-3, and MOC-31 and less useful are alpha fetoprotein (AFP), TTF-1, cytokeratin (CK) 8, CK18, CD10 and CD34 (13).

In the present study, HepPar1 and CD 10 are very useful in the adjunct diagnosis of HCC. Hep Par 1 has emerged as the most sensitive and specific (both >80%) immunohistochemical marker for well differentiated HCC and negative in poorly differentiated and sclerosing HCC. CD10 shows canalicular pattern of staining which is specific for HCC and sensitivity for well and moderately differentiated HCC (13).

After a diagnosis of HCC has been confirmed; staging, liver function, performance status and comorbid conditions are main factors on which treatment depends. At early stage, tumors are more amenable to potentially curative treatments, such as resection, ablation, and liver transplantation. In advanced stage targeted therapy (sorafenib) or chemotherapy may be options. 

Sorafenib is one of the first line drug, which has been extensively used in metastatic, and inoperable hepatocellular carcinoma (14). Sorafenib, an orally available multikinase inhibitor that inhibits cell surface tyrosine kinase receptors like VEGFR-2/3, PDGFR-Beta, B-raf, C-raf, FLT-3 and RET1 (15). Sorafenib inhibits cell growth in a dose and time dependent manner by altering the expression of genes involved in angiogenesis, apoptosis and transcriptional regulation. Toxicity profile of sorafenib is mainly skin rash, liver toxicity, fatigue and vascular toxicity.

In conclusion HCC metastasis to right cervical lymph nodes, although rarely, does occur. We conclude hereby that metastasis of HCC should be included in the differential diagnosis of right sided cervical lymphadenopathy even in the absence of signs and symptoms of liver disease.

We believe, it is worth having to report of such unusual entity as and when they occur in order to document their natural history and plan better treatment strategies for best possible outcome.
